# Fermented Chinese Herbs Improved Egg Production, Egg Shell Quality, and Egg Yolk Cholesterol of Laying Hens by Regulating Estrogen, Lipid Metabolism, and Calcium Metabolism

**DOI:** 10.3390/ani15213073

**Published:** 2025-10-23

**Authors:** Xinyu Liu, Yue He, Yuhan Cao, Xin Wang, Ye Yang, Jiao Song

**Affiliations:** 1 College of Animal Science and Technology, Yangtze University, Jingzhou 434025, China; 15572180927@139.com (X.L.); 13177112816@163.com (Y.H.); 15756303545@163.com (Y.C.); yangyecaas@sina.com (Y.Y.); 2 College of Life Science, Yangtze University, Jingzhou 434025, China; wx11064012@126.com

**Keywords:** fermented Chinese herbs, laying hen, eggshell quality, yolk cholesterol, calcium metabolism

## Abstract

**Simple Summary:**

Eggshell quality and egg cholesterol content are important factors determining consumer acceptability and egg production efficiency. Use of Chinese herbal compounds is an efficient nutritional regulation means of modulating egg quality. In this study, we evaluated the effects of a specific Chinese herbal compound on egg production, egg shell quality, and egg yolk cholesterol by assessing its ability to regulate estrogen, lipid metabolism, and calcium metabolism. The results indicate that diet supplementation with a fermented Chinese herbal compound may improve egg quality by regulating reproductive hormones, lipid metabolism, and calcium metabolism, providing a theoretical basis for the practice of using traditional Chinese herbs.

**Abstract:**

The present study investigated the effects of fermented Chinese herbal (FCH) compounds on the egg production, egg shell quality, and egg yolk cholesterol of laying hens. A total of 1260 Hy-Line pink laying hens, 34 weeks old, were randomly divided into three groups, with six replicates per group and 70 hens per replicate, as follows: the control group (CON group) was fed a diet without FCH compounds, and the 2% FCH group and the 3% FCH group were fed a diet supplemented with 2% FCH and 3% FCH, respectively. The results show that the FCH compound significantly increased the laying rate compared to the CON group (*p* < 0.05). Analyses of the serum biochemical indices showed that supplementation with FCH compound significantly decreased the levels of total cholesterol (TC), total triglyceride (TG), high-density lipoprotein cholesterol (HLDL-c), low-density lipoprotein cholesterol (LDL-c), very-low-density lipoprotein cholesterol (VLVL-c), aspartate transaminase (AST), and alanine aminotransferase (ALT) (*p* < 0.05) and increased the serum total bile acids, follicle-stimulating hormone (FSH), luteinizing hormone (LH), and 17-β-Estradiol (E2) levels (*p* < 0.05). The FCH group significantly increased the activity of superoxide dismutase (SOD) and total antioxidant capacity and decreased malondialdehyde (MDA) levels in the liver and uterus compared to the CON (*p* < 0.05). FCH supplementation was also associated with improved egg quality, seen through factors including enhanced yolk color, albumen height, Haugh unit score, eggshell strength, and thickness and reduced egg breaking rate and TC and TG contents in egg yolk. The gene expression analyses showed that FCH supplementation significantly increased the calcium metabolism-related gene expression (*CaBP-D_28k_*, *NCX*, *VDR*, *CYP*_27_*B*_1_, *OPN*, *PMCA*, *CA_2_*) in duodenum, kidney, and uterus tissues compared to the CON group (*p* < 0.05). FCH significantly repressed *FAS* and *HMGCR* mRNA expression and enhanced *CYP7A1* mRNA expression in the liver (*p* < 0.05). These results indicate that diet supplementation with FCH compounds may improve egg quality by regulating reproductive hormones, lipid metabolism, and calcium metabolism.

## 1. Introduction

Eggshell quality is an important index to evaluate egg quality, which directly impacts the egg qualification rate, hatchability, and market value. Especially with the continuous application of mechanized production in egg collection and packaging, an increase in cracked eggs poses a great threat to laying production. A study showed that eggshell calcium (Ca) metabolism indirectly determines eggshell quality [1]. Eggshell Ca supply and estrogenic regulation effects are the main factors contributing to eggshell calcification and eggshell quality. Ca derived from intestinal absorption, regulated by vitamin D receptor (VDR) and re-absorbed in the kidney, is transported to the uterus and calcified to form eggshells. This process is regulated by osteopontin (OPN) in the eggshell gland [2]. Ca transportation is regulated by Ca transporters, such as plasma membrane calcium-ATPase (PMCA), sodium-calcium exchanger (NCX), and calbindin-D_28k_ (CaBP-D_28k_) [3]. Moreover, several reproductive hormones, mainly including follicle-stimulating hormone (FSH), luteinizing hormone (LH), and 17-β-Estradiol (E2), also have an important regulatory effects on Ca metabolism [4].

Egg cholesterol content in egg yolk is another critical value for evaluating egg quality. Although eggs are rich in many essential nutrients needed for human health, such as high-quality protein, minerals, and vitamins, the high concentration of cholesterol is a risk for human health, especially hypercholesteremia and cardiovascular disease. The cholesterol content is approximately 200~275 mg in one large egg yolk, which is close to the recommended daily cholesterol intake according to the American Heart Association [5]. A study showed that 100 mg of egg cholesterol increased total cholesterol [6]. Therefore, excessive egg consumption is generally positively correlated with atheroscle rotic cardiovascular disease [6]. In addition to liver lipid metabolism, the cholesterol content in egg yolk is also regulated by cholesterol biosynthesis and transformation in the livers of laying hens. Cholesterol synthesized in the liver is transported to the ovaries in the form of very-low-density lipoprotein cholesterol (VLDL) via the bloodstream and participates in the formation of egg yolks [7]. The rate-limiting enzyme of cholesterol biosynthesis is 3-hydroxy-3-methylglutaryl-CoA reductase (HMGCR). The key enzyme regulating the transformation of cholesterol to bile acids is cholesterol 7α hydroxylase (CYP7A1) in the liver [8].

Recent studies have indicated that Chinese herbs and their extracts have the ability to improve laying rates, egg quality, and eggshell quality and to regulate lipid metabolism and estrogen levels [9,10,11]. In China, several specific Chinese herbs, such as *Leonurus japonicus* (Chinese motherwort), *Jujube*, *Artemisia argyi* (*A. argyi*), and *Radix isatidis*, have been used for thousands of years to regulate hormone metabolism, enhance immune effects, and improve blood circulation [12,13,14,15]. Additionally, numerous studies have shown that these Chinese herbs have exhibited regulating effects for inhibiting hepatic lipid synthesis and reducing fat deposition [5,16,17,18]. These herbs contain a variety of active ingredients, especially including alkaloids, flavonoids, and diterpenes, which have been reported to regulate estrogen metabolism and anti-oxidative stress effects [11,19]. Practical experience with traditional Chinese herbs has confirmed that Chinese herb compounds exhibit greater biological effects than individual herbs [20,21]. To date, the combination of *Leonurus japonicus*, *jujube*, *Artemisia argyi* (*A. argyi*), and *Radix isatidis* has not been studied in laying hens. Therefore, based on traditional Chinese herbal theory, the present study utilizes herb compounds to modulate antioxidant properties and improve egg quality and cholesterol content.

The results show that processing technology has an important influence on Chinese herbal pharmacological activities. Because Chinese herbs are protected by a hard cell wall, their application as additives results in low utilization rates and poor palatability, which reduces their regulatory effects [4]. Numerous studies have shown that probiotic fermentation and enzymatic methods can effectively enhance the utilization efficiency of Chinese herbs [22,23,24]. Previous findings indicated that fermented Chinese herbal medicines significantly improved laying rates, egg quality, and eggshell strength, and increased plasma estradiol levels [25].

Therefore, the purpose of the present study was to clarify the regulatory influence of fermented Chinese herb compounds on egg production, eggshell quality, yolk cholesterol, and estrogenic effects.

## 2. Materials and Methods

Experimental protocols were performed in accordance with the guidelines of the Ethics Committee of Yangtze University (No. DKYB202400412) in China for the humane care and use of animals in research.

### 2.1. Preparation of Fermented Chinese Herbs

Fermented Chinese Herbs (FCH) were supplied by Wuhan Dabeinong Science and Technology Park (Wuhan, China). Briefly, the formulation of Chinese herbs consisted of a combination of 15% *motherwort*, 8% *Artemisia argyi*, 11% *radix isatidis*, and 8% *jujube powder*, with 28% corn, 14% wheat bran, and 16% soybean meal as the fermentation carriers. The fermentation bacterial agent included *Bacillus subtilis* 1.0 × 10^10^ CFU/mL, *Bacillus coagulans* 3.0 × 10^10^ CFU/mL, *Enterococcus faecalis* 2.4 × 10^10^ CFU/mL, *Lactobacillus plantarum* 4.0 × 10^11^ CFU/mL, and *Saccharomyces cerevisiae* 4.5 × 10^10^ CFU/mL. The fermentation substrate was processed to 50% moisture content, then inoculated with 4% of microbial combination liquid. Subsequently, fermentation was carried out in a fermentation bag with a one-way breather valve for 7 d to prepare FCH. Then, the FCH (with water content of about 38~40%) was added to feed. The main approximate nutrient contents of the FCH, measured by AOAC [26], are shown in Table 1.

### 2.2. Experimental Animals and Design

A total of 1260 healthy Hy-Line laying hens (34-week-old) were randomly divided into three groups, with 6 replicates per group and 70 hens per replicate: the CON group, the 2% FCH group, and the 3% FCH group. The hens in the 2% FCH group and the 3% FCH group were fed the diet supplemented with 2% FCH and 3% FCH, respectively. The diet was prepared according to NRC (1994) [27] to meet the nutrient needs of laying hens (Table 2). The experiment lasted for 8 weeks. After one week of adaptation, all laying hens were fed the assigned experimental diets for 7 weeks. Artificial light was used throughout the house of the test laying hens. A total of 3~4 hens were housed in each metal cage (48 × 36 × 40 cm), with an adjoining cage consisting of 70 hens per replicate. The temperature was kept at 24~26 °C, and the relative humidity was kept at 50~60%. Feeds were supplied ad libitum in mash.

### 2.3. Sample Collection

At the end of the experiment, two hens per replicate were randomly selected and slaughtered for sample collection. The serum was separated from blood with centrifugation at 1000 g/min for 15 min and stored at −20 °C to analyze the serum biochemical indexes, serum hormone, and bile acids. Liver, duodenal tissue mucosa, kidney, and uterus samples (one hen per replicate) were removed, placed into tubes, snap-frozen in liquid nitrogen, and then stored at −80 °C until analysis. Meanwhile, 4 eggs per replicate were selected to determine egg quality, eggshell, and egg yolk indices on the 28th and 56th days of the experiment.

### 2.4. Performance

During the experiment, the number of eggs produced, egg weight, and feed consumption were recorded daily. At the time of egg collection, all eggs were classified as normal, cracked, or soft. Average daily egg weight (ADEW), average daily feed intake (ADFI), feed–egg ratio, egg laying rate, cracked shell egg rate, and soft shell egg rate were calculated in replicate units based on daily records.

### 2.5. Measurement of Egg Quality

A high-precision digital egg tester (DET-6 000, Beijing Bulad Technology Development Co., Ltd., Beijing, China) was used to measure the yolk color, albumen height, and Haugh unit score. The eggshell strength was determined by the EFG-0503 egg shell force gauge (Robotmation, Tokyo, Japan). The eggshell thickness was determined by the SD201 digital display thickness gauge (Shenzhen Yuanhengtong Technology Co., Ltd., Shenzhen, China) by calculating the average thickness of the air cell, equator, and sharp end segments. Eggshell color was measured using a TS20 color colorimeter (Shenzhen 3nh Technology Co., Ltd., Shenzhen, China).

### 2.6. Ca Determination in Eggshell

After peeling off the inner membrane, the eggshell was crushed into powder and oven-dried at 103 °C for 2 h. Then, the powder was ashed at 600 °C in a muffle furnace for 3 h. Finally, the content of Ca was measured by potassium permanganate titration [28].

### 2.7. Assay of Serum Biochemical Indices, Serum Hormone, and Serum Bile Acids

The serum biochemical indices were determined with ELISA using a Hitachi 7600 automated biochemical analyzer (Hitachi, Tokyo, Japan). Assay kits for aspartate transaminase (AST, C010-2-1), alanine aminotransferase (ALT, C009-2-1), triglyceride (TG, F001-1-1), total cholesterol (TC, F002-1-1), high-density lipoprotein cholesterol (HDL-c, A112-1-1), low-density lipoprotein cholesterol (LDL-c, A113-2-1), very-low-density lipoprotein cholesterol (VLDL-c, H249-1-2), and total bile acid (TBA, E003-1-1) were purchased from the Nanjing Jiancheng Bioengineering Institute (Nanjing, China). The determination was carried out following the manufacturer’s instructions.

Serum hormone levels of FSH (H101-1-2) and LH (H206-1-2) were determined by ELISA kits (Nanjing Jiancheng Bioengineering Institute, Nanjing, China). Serum concentrations of E2 (TW15203) were determined by ELISA kits (Shanghai Tongwei Biotechnology Co., Ltd., Shanghai, China).

### 2.8. Measurement of Antioxidant Enzyme Activity in the Liver and Uterus

The total antioxidant capacity (T-AOC), superoxide dismutase (SOD) activity, and malondialdehyde (MDA) content in the liver and uterus were determined by an assay kit (Nanjing Jiancheng Bioengineering Institute, Nanjing, China).

### 2.9. Measurements of TC and TG in Liver and Egg Yolk and Liver TBA

The TC (F002-1-1), TG (F001-1-1) in the liver and egg yolk were determined with commercial assay kits (Nanjing Jiancheng Bioengineering Institute, Nanjing, China) and performed using a Hitachi 7600 automated biochemical analyzer (Hitachi, Tokyo, Japan). The liver TBA (MAK309) was determined with assay kits (Sigma-Aldrich Life Science, Shanghai, China).

### 2.10. Gene Expression of Cholesterol Metabolism in Liver and Ca Metabolism in Kidney, Duodenum and Uterus

Methods of RNA extraction and qRT-PCR of samples were followed the procedures described in our previous research [29]. Total RNA was extracted from duodenum, liver, and uterus samples using Trizol reagent (Thermo Fisher Scientific, New York, NY, USA). First-strand cDNA synthesis was performed using the FastKing cDNA first-strand synthesis kit (Beijing Tiangen Biotechnology Co., Ltd., Beijing, China). The primers used in this study were designed using the Primer 5.0 software package (Table 3). The *GAPDH* gene was used as an internal reference to standardize the target gene levels, and the relative mRNA expression of the target genes was calculated as a ratio to the *GAPDH* gene using the 2^−ΔΔCT^ method.

### 2.11. Statistical Analysis

Statistical analyses were performed using SAS V9.4. Data were analyzed with one-way ANOVA using Duncan’s test. Orthogonal polynomial contrasts were also used to evaluate the linear and quadratic effects of FCH levels. Statistical differences were considered significant at *p* < 0.05.

## 3. Results

### 3.1. FCH and Egg Production Performance

The results of egg production performance are presented in Table 4. Compared with the CON group, FCH had no significant effect on the ADFI, ADEW, or feed/egg ratio (*p* < 0.05). FCH significantly increased the laying rate in the final trial compared to the CON group (*p* < 0.05). The 2% FCH group had a higher laying rate in the final trial than the 3% FCH group (*p* < 0.05).

### 3.2. FCH and Serum Biochemical Indices

The results of serum biochemical traits are presented in Table 5. Compared with the CON group, FCH decreased the levels of TC, TG, HDL-C, LDL-C, VLDL-C, AST, and ALT (*p* < 0.05) and increased the serum total bile acid contents (*p* < 0.05).

### 3.3. FCH and Serum Hormone

The results of serum hormone are presented in Table 6. Compared with the CON, FCH increased the levels of FSH, LH, and E2 (*p* < 0.05). The 3% FCH group had higher levels of FSH, LH, and E2 than the 2% FCH group (*p* < 0.05).

### 3.4. FCH and Antioxidant Enzyme Activity

The results of antioxidant traits are presented in Table 7. Compared with the CON, FCH significantly increased the activities of SOD and T-AOC and decreased the MDA levels in the liver and uterus (*p* < 0.05). The 3% FCH group had higher SOD and T-AOC activities in the liver and uterus than the 2% FCH group (*p* < 0.05).

### 3.5. FCH and Egg Quality Traits

The results of egg quality are shown in Table 8. Compared with the CON, FCH significantly increased the yolk color, albumen height, and Haugh unit score (*p* < 0.05). The analysis of egg shell showed that FCH significantly increased the eggshell strength and thickness and decreased the egg breaking rate compared to the CON group (*p* < 0.05). The 3% FCH group had stronger eggshell color than the CON group and the 2% FCH group (*p* < 0.05). FCH decreased the soft shell egg rate, but the difference was not significant compared to the CON group (*p* < 0.05).

### 3.6. Ca Deposition in Eggshell and Ca Metabolism-Related Gene Expression

**Figure 1:**
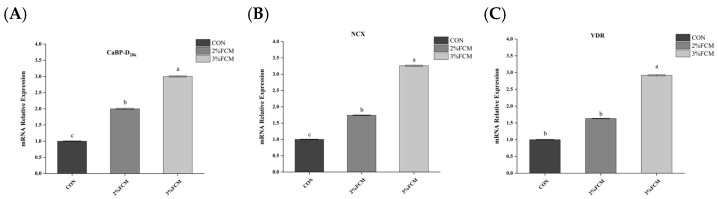
The effects of FCH on Ca metabolism-related gene expression in duodenum. ^a,b,c^ Different superscripts above bars indicate significant differences (*p* < 0.05). Note: CON, a basal diet; 2% FCH, 2% fermented Chinese herbs; 3% FCH, 3% fermented Chinese herbs; (**A**) *CaBP-D_28k_*, calcium transporter calbindin-D_28k_; (**B**) *NCX*, sodium-calcium exchanger; (**C**) *VDR*, vitamin D receptor.

**Figure 2:**
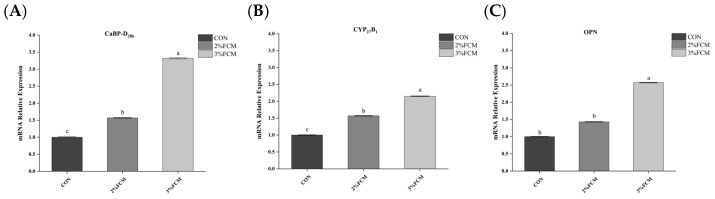
The effects of FCH on Ca metabolism-related gene expression in kidney (*n* = 6). ^a,b,c^ Different superscripts above bars indicate significant differences (*p* < 0.05). Note: CON, a basal diet; 2% FCH, 2% fermented Chinese herbals; 3% FCH, 3% fermented Chinese herbals; (**A**) *CaBP-D_28k_*, calcium transporter calbindin-D_28k_; (**B**) *CYP_27_B_1_*, recombinant cytochrome p450 27 B1; (**C**) *OPN*, osteopontin.

**Figure 3:**
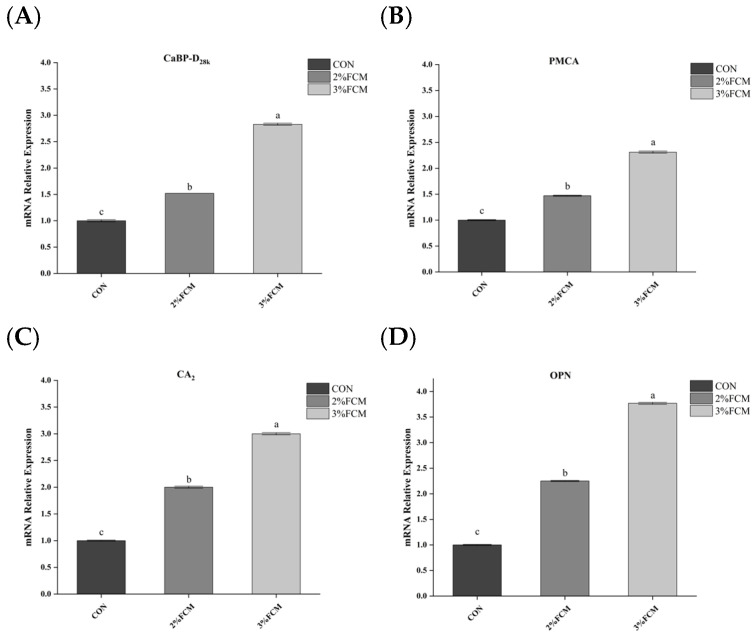
The effects of FCH on Ca metabolism-related gene expression in uterus (*n* = 6). ^a,b,c^ Different superscripts above bars indicate significant differences (*p* < 0.05). Note: CON, a basal diet; 2% FCH, 2% fermented Chinese herbals; 3% FCH, 3% fermented Chinese herbals; (**A**) *CaBP-D2_8k_*, calcium transporter calbindin-D_28k_; (**B**) *PMCA*, plasma membrane calcium-ATPase; (**C**) *CA_2_*: carbonic anhydrase; (**D**) *OPN*, osteopontin.

As shown in Table 8, FCH significantly increased eggshell Ca deposition compared to the CON group (*p* < 0.05). The Ca metabolism-related gene expression in the duodenum, kidney, and uterus tissues showed that FCH significantly increased the mRNA expressions of *CaBP-D_28k_*, *NCX*, the *VDR* in the duodenum (Figure 1), *CaBP-D_28k_*, *CYP*_27_*B*_1_, and *OPN* in the kidney (Figure 2), and *CaBP-D_28k_*, *PMCA*, *CA2*, and *OPN* in the uterus tissues (Figure 3) compared to the CON group (*p* < 0.05).

### 3.7. Cholesterol and Triglyceride Deposition in Liver and Egg Yolk and Cholesterol Metabolism-Related Gene Expression

**Figure 4:**
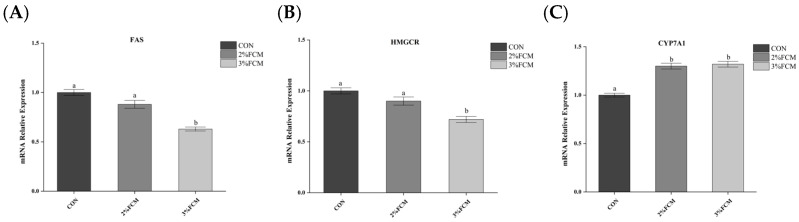
The effects of FCH on cholesterol metabolism-related gene expression in liver (*n* = 6). ^a,b^ Different superscripts above bars indicate significant differences (*p* < 0.05). Note: CON, a basal diet; 2% FCH, 2% fermented Chinese herbals; 3% FCH, 3% fermented Chinese herbals; (**A**) *FAS*, fatty acid synthetase; (**B**) *HMGCR*, β-hydroxy-β-methylglutaryl-coenzyme reductase; (**C**) *CYP7A1*, cholesterol-7α-hydroxylase.

As shown in Table 9, compared with the CON group, FCH reduced the TC and TG contents in the liver and egg yolk and increased liver bile acid levels (*p* < 0.05). As shown in Figure 4, FCH significantly down-regulated the mRNA expressions of *FAS* and *HMGCR* and up-regulated the mRNA expression of *CYP7A1* (*p* < 0.05).

## 4. Discussion

In general, the present study with laying hens showed that dietary supplementation with FCH enhanced egg production and improved egg quality, mainly by enhancing eggshell quality and decreasing egg yolk cholesterol content. The results also reveal that the FCH exhibited positive effects on serum estrogen, liver lipid metabolism, and oxidative stress in laying hens.

Egg shell quality is a major concern for egg producers, as it directly affects the egg appearance and cracked rate in the process of packaging and transportation [30]. The evaluation of eggshell quality mainly includes eggshell strength, eggshell thickness, eggshell color degree, egg breaking rate, and soft shell egg rate. Eggshells are mainly composed of calcium carbonate. Therefore, Ca metabolism determines eggshell calcification and quality during formation [21]. Ca derived from intestinal absorption and re-absorption in the kidney is transported to the uterus and calcified to form eggshell. Ca transportation is regulated by calcium transporters, such as PMCA, NCX, and CaBP-D_28k_ [31]. CaBP-D_28k_ is the main calcium-binding protein in laying hens, involved in calcium metabolism in the duodenum, kidney, and eggshell gland [2]. VDR is involved in the uptake of active vitamin D3. Meanwhile, active vitamin D3 is also regulated by CYP27B1, which promotes 1,25(OH)_2_D_3_ activity [32,33]. NCX takes part in the transport of Ca^2+^ from the duodenum into the blood. Eggshell calcification is regulated by the glycoprotein OPN [2]. Therefore, the expression of genes related to eggshell calcification is an important factor affecting eggshell quality. This experimental study found that FCH significantly increased the relative mRNA expressions of *NCX*, liver *CaBP-D_28k_*, and uterine *PMCA*, *CA_2_*, and *OPN*. The results indicate that FCH improved eggshell quality by regulating calcium metabolism.

A study showed that a Chinese herb mixture regulated eggshell Ca metabolism by modulating sex hormone levels [11]. Eggshell calcification is regulated by several estrogen, such as FSH, LH, E2, and gonadotropin-releasing hormone (GnRH). Specific Chinese herbs, such as *Leonurus japonicus*, exhibit the function of regulating sex hormone levels and egg quality [34]. The main active compounds (flavonoids and alkaloids) in *Leonurus japonicus* and A. *argyi* were reported to regulate estrogen metabolism [34]. The present study showed that FCH increased the laying rate and the levels of FSH, LH, and E2. FSH and LH play important roles in regulating follicular maturation and reproductive function [28]. E2 regulates shell calcification via calcium metabolism in poultry [35]. Extensive studies have also shown that higher estrogen concentrations in serum are closely positively related to egg production and eggshell quality [28,36].

Egg cholesterol content is a major concern for consumers. Excessive egg consumption contributes to high-cholesterol-related diseases, such as atheroscle rotic cardiovascular disease [6]. It is an important objective in laying hen production to reduce cholesterol content in eggs. Egg yolk cholesterol comes from the liver. Therefore, the egg cholesterol content is affected by cholesterol synthesis, transportation, and degradation. Cholesterol is synthesized by the critical rate-limiting enzyme HMGCR in the liver. With the action of lipoprotein VLDL, the cholesterol is transported to the ovaries and takes part in the formation of egg yolks [7]. Cholesterol content is also reduced by breaking down to bile acid with the action of *CYP7A1* mRNA expression. Therefore, egg cholesterol content is affected by the expressions of *HMGCR* and *CYP7A1* and the VLDL level. The present study showed that FCH decreased the serum VLDL-C level and *HMGCR* mRNA expression and increased the *CYP7A1* mRNA expression, which resulted in a decrease in egg cholesterol.

Numerous studies have shown that Chinese herbs have regulatory effects on hepatic lipid metabolism in laying hens [5,16,17,18]. *Leonurus japonicus* exhibited inhibiting effects on hepatic lipid synthesis [17]. *Artemisia argyi* exerted depression effects on lipid metabolism [16]. *Jujube* and *Radix isatidis* also repressed fat deposition and down-regulated the expressions of the main adipogenic transcription factors (such as *PPARγ*) via the AMPK signaling pathway [5,18]. The present research also showed that the Chinese herb compounds inhibited *FAS* and *HMGCR* expression and decreased the TC and TG contents in the liver and egg yolk. Additionally, a previous study showed that estrogen plays an important role in lipid metabolism in the liver of hens. Estrogen decreased the expressions of genes involved in lipid metabolism and reduced lipid deposition [37]. This study also indicated that the Chinese herb compounds decreased the liver lipid contents by enhancing the estrogen FSH, LH, and E2 levels.

Oxidative stress is a key factor affecting egg production, egg quality, and lipid metabolism in laying hens [38,39]. Multiple studies have shown that excess ROS can induce follicular atresia and repress follicular growth and ovulation, which leads to drops in egg production [38]. Moreover, oxidative stress increases the occurrence of liver impairment, resulting in fatty liver syndrome [38]. The present study showed that FCH increased the activities of SOD and T-AOC and decreased MDA levels in the liver and uterus. Meanwhile, FCH also increased the liver total bile acid (TBA) contents and decreased the activities of AST and ALT. AST and TBA are critical indices for evaluating liver health status [39]. Multiple studies have indicated that the natural activity components (flavonoids, terpenoids, polysaccharides, and polyphenols) of the present Chinese herb compounds exhibit important roles in antioxidant properties [13,14,40]. The present study also indicated that CFM increased egg production and decreased egg cholesterol content by enhancing antioxidant properties in the liver and uterus.

## 5. Conclusions

This research demonstrated that dietary supplementation with FCH containing *Leonurus japonicus*, *Jujube*, *Artemisia argyi*, and *Radix isatidis* increased egg laying performance and improved egg quality, mainly including yolk color, egg yolk cholesterol content, and eggshell strength and thickness. These beneficial effects were associated with reproductive hormones, antioxidant properties, lipid metabolism, and calcium metabolism induced by FCH. In summary, these findings suggested that the specific FCH mixture used was a good feed additive for laying hens.

## Figures and Tables

**Table 1:** Main nutritional components of traditional Chinese herbs (air-dry basis).

Nutrients ^1^	Unfermented Chinese Herbs	Fermented Chinese Herbs
DE (MJ/kg)	5.74	5.86
Crude protein (%)	11.54	11.70
Crude fiber (%)	11.96	11.78
Ash (%)	8.99	9.00
Calcium (%)	0.13	0.15
Total phosphorus (%)	0.42	0.46

Note: DE, digestible energy. ^1^ The nutrient contents were measured values.

**Table 2:** The composition and nutrient levels of diets (air-dry basis) %.

Item	Treatment		
	CON	2% FCH	3% FCH
Ingredient (%)			
Corn	62.28	62.18	61.20
Soybean meal (CP 45%)	21.90	22.00	21.88
Soybean oil	0.50	0.50	0.50
Wheat bran	1.80	1.80	1.80
Rice bran	3.00	1.00	1.00
Limestone	8.00	8.00	8.00
FCH	0.00	2.00	3.00
CaHPO4·2H_2_O	1.30	1.30	1.40
50% choline chloride	0.12	0.12	0.12
DL-methionine	0.10	0.10	0.10
Premix feed ^(1)^	1.00	1.00	1.00
Total	100.00	100.00	100.00
Nutrient levels (%) ^(2)^			
Metabolic energy (MJ/kg)	11.50	11.50	11.50
Crude protein	16.30	16.30	16.30
Total phosphorus	0.50	0.50	0.50
Available phosphorus	0.32	0.32	0.32
Calcium	3.60	3.60	3.61
Lysine	0.83	0.83	0.83
Methionine	0.34	0.34	0.34

Note: CON, a basal diet; 2% FCH, 2% fermented Chinese herbs; 3% FCH, 3% fermented Chinese herbs. ^(1)^ The premix provided the following per kg of diets: vitamin A (retinol acetate), 8000 IU; vitamin D_3_, 2000 IU; vitamin E (DL-α-tocopherol acetate), 15 IU; vitamin B_1_, 3 mg; vitamin B_2_, 9 mg; vitamin B_6_, 6 mg; vitamin B_12_, 0.03 mg; nicotinic acid 60 mg; calcium pantothenate, 18 mg; folic acid, 1.5 mg; biotin, 0.36 mg; choline chloride, 500 mg; Fe, 60 mg; Zn, 80 mg; Cu, 8 mg; Mn, 600 mg; I, 0.48 mg; Se, 0.06 mg. ^(2)^ The levels of crude protein and calcium were measured values, while the others were calculated values.

**Table 3:** Primers of genes.

Genes	Primer Sequence (5′ → 3′)	Gene Bank ID
*CaBP-D* *_28k_*	F: AGACTTCATGCAGACATGGAGA	NM_205513.2
	R: CTTGATCGTACATCTGAAAGGC	
*VDR*	F: GATGGCAACGCACTCC	XM_046934193.1
	R: CTGCCGTCAGATCCCT	
*PMCA*	F: CATGAGTTCAGCATCACCGG	XM_046929909.1
	R: TTTTCCACCAAGCACGTCAG	
*NCX*	F: GGGAAGGCTTGCTCAC	XM_046913144.1
	R: CTACCTCCAACACCAG	
*OPN*	F: AGGTGGACGGAGGAGACA	NM_204535.5
	R: ACGGGTGACCTCGTTGTT	
*CYP* *_27_* *B* *_1_*	F: AACATTTCACGCAATCCACA	NM_001396287.1
	R: AATGGCACAGATGGTGTCAA	
*CA*_2_	F: CAACAACGGGCACTCC	NM_205317.2
	R: TGGCATTCCCTACCTT	
*CYP7A1*	F: GATCTTCCCAGCCCTTGTGG	AY700578
	F: AGCCTCTCCCAGCTTCTCAC	
*FAS*	F: CAATGGACTTCATGCCTCGGT	J04485
	R: GCTGGGTACTGGAAGACAAACA	
*HMGCR*	F: ATGTCAGGAGTGCGACAACT	XM_046934671.1
	R: CGTCCTTCACGACTCTCTCG	

Note: *CaBP-D_28k_*: calcium transporter calbindin-D_28k_; *VDR*: vitamin D receptor; *PMCA*: plasma membrane calcium-ATPase, *NCX*: sodium-calcium exchanger, *OPN*: osteopontin; *CYP_27_B_1_*: recombinant cytochrome P450 27 B1; *CA_2_*: carbonic anhydrase; *CYP7A1*: cholesterol-7α-hydroxylase; *FAS*: fatty acid synthetase; *HMGCR*: β-hydroxy-β-methylglutaryl-coenzyme reductase.

**Table 4:** Effects of FCH on performance in laying hens.

Items	Treatment			*p*-Value
	CON	2% FCH	3% FCH	
ADFI (g/hen)	122.65 ± 6.37	123.06 ± 5.73	123.19 ± 6.28	0.911
ADEW (g/hen)	61.84 ± 2.18	62.43 ± 3.24	62.08 ± 3.37	0.383
FCR	1.98 ± 0.02	1.97 ± 0.03	1.98 ± 0.02	0.894
Laying rate of initial trial (%)	81.79 ± 2.65	81.59 ± 3.18	81.83 ± 3.52	0.972
Laying rate of final trial (%)	82.74 ± 3.37 ^c^	89.96 ± 2.84 ^a^	86.87 ± 2.17 ^b^	<0.001

^a,b,c^ values within a row with different superscripts mean significant difference (*p* < 0.05). Note: Data are expressed as the means ± SE (standard error), *n* = 420. CON = basal diet; 2% FCH = 2% fermented Chinese herbs; 3% FCH = 3% fermented Chinese herbs; ADFI = average daily feed intake; ADEW = average egg weight; FCR = g feed/g egg ratio.

**Table 5:** Effects of FCH on serum biochemical indices in laying hens.

Items	Treatment			*p*-Value
	CON	2% FCH	3% FCH	
TC (mmol/L)	2.65 ± 0.12 ^a^	2.19 ± 0.11 ^b^	1.76 ± 0.13 ^c^	0.018
TG (mmol/L)	5.36 ± 0.15 ^a^	4.25 ± 0.12 ^b^	3.38 ± 0.13 ^c^	0.015
HDL-C (mmol/L)	2.15 ± 0.11 ^a^	1.85 ± 0.08 ^b^	1.73 ± 0.11 ^c^	0.013
LDL-C (mmol/L)	0.89 ± 0.02 ^a^	0.74 ± 0.03 ^b^	0.65 ± 0.01 ^c^	0.027
VLDL-C (mmol/L)	0.78 ± 0.03 ^a^	0.65 ± 0.02 ^b^	0.59 ± 0.02 ^c^	0.033
AST (U/L)	20.35 ± 1.57 ^a^	18.26 ± 1.38 ^b^	18.03 ± 1.21 ^c^	0.025
ALT (U/L)	9.16 ± 0.18 ^a^	8.12 ± 0.14 ^b^	7.34 ± 0.15 ^c^	0.016
Bile acids (µmol/L)	5.12 ± 0.12 ^c^	6.75 ± 0.16 ^b^	7.23 ± 0.13 ^a^	0.014

^a,b,c^ values within a row with different superscripts mean significant differences (*p* < 0.05). Note: Data are expressed as the means ± SE (standard error), *n* = 12. CON = basal diet; 2% FCH = 2% fermented Chinese herbals; 3% FCH = 3% fermented Chinese herbals; TC = total cholesterol; TG = triglyceride; HDL-c = high-density lipoprotein cholesterol; LDL-c = low-density lipoprotein cholesterol; VLDL-c = very-low-density lipoprotein cholesterol; AST = aspartate transaminase; ALT = alanine aminotransferase.

**Table 6:** Effects of FCH on serum hormone indexes in laying hens.

Items	Treatment			*p*-Value
	CON	2% FCH	3% FCH	
FSH (ng/L)	3.17 ± 0.05 ^c^	4.36 ± 0.03 ^b^	4.91 ± 0.02 ^a^	0.013
LH (IU/L)	8.25 ± 0.09 ^c^	10.37 ± 0.12 ^b^	11.28 ± 0.15 ^a^	0.016
E2 (pg/mL)	510.09 ± 10.36 ^c^	852.61 ± 12.41 ^b^	976.18 ± 15.72 ^a^	<0.001

^a,b,c^ values within a row with different superscripts mean significant differences (*p* < 0.05). Note: Data are expressed as the means ± SE (standard error), *n* = 12. CON = basal diet; 2% FCH = 2% fermented Chinese herbs; 3% FCH = 3% fermented Chinese herbs; E2 = 17-β-Estradiol; FSH = follicle-stimulating hormone; LH = luteinizing hormone.

**Table 7:** Effects of FCH on antioxidant capacity in liver and egg yolk of laying hens.

Items		Treatment			*p*-Value
		CON	2% FCH	3% FCH	
liver	SOD (U/g)	0.59 ± 0.01 ^c^	0.67 ± 0.01 ^b^	0.81 ± 0.02 ^a^	0.015
	T-AOC (U/mg)	256.85 ± 9.97 ^c^	373.88 ± 11.35 ^b^	474.32 ± 12.48 ^a^	<0.001
	MDA (nmol/mg)	5.11 ± 0.17 ^a^	4.13 ± 0.12 ^b^	3.46 ± 0.08 ^b^	0.002
Uterus	SOD (U/g)	24.19 ± 0.25 ^c^	31.84 ± 0.18 ^b^	35.46 ± 0.23 ^a^	<0.001
	T-AOC (U/mg)	10.36 ± 0.17 ^c^	16.25 ± 0.13 ^b^	18.37 ± 0.16 ^a^	0.019
	MDA (nmol/mg)	2.14 ± 0.02 ^a^	1.57 ± 0.01 ^b^	1.26 ± 0.01 ^c^	0.014

^a,b,c^ values within a row with different superscripts mean significant differences (*p* < 0.05). Note: Data are expressed as the means ± SE (standard error), *n* = 6. CON = basal diet; 2% FCH = 2% fermented Chinese herbs; 3% FCH = 3% fermented Chinese herbs; T-AOC = total antioxidant capacity; SOD = superoxide dismutase; MDA = malondialdehyde.

**Table 8:** Effects of FCH on egg quality in laying hens.

Items	Treatment			*p*-Value
	CON	2% FCH	3% FCH	
Yolk color	13.27 ± 0.12 ^b^	13.69 ± 0.15 ^b^	14.27 ± 0.04 ^a^	0.017
Albumen height (mm)	6.89 ± 0.03 ^b^	7.81 ± 0.03 ^a^	8.02 ± 0.05 ^a^	0.006
Haugh unit score	83.13 ± 1.37 ^b^	87.59 ± 1.25 ^a^	89.45 ± 1.29 ^a^	0.021
Eggshell strength (N)	44.5 ± 1.43 ^b^	52.20 ± 1.36 ^a^	55.24 ± 1.52 ^a^	0.006
Eggshell thickness (mm)	0.36 ± 0.02 ^b^	0.39 ± 0.01 ^a^	0.40 ± 0.01 ^a^	0.025
Eggshell color	74.69 ± 1.38 ^b^	75.09 ± 1.55 ^b^	76.58 ± 1.29 ^a^	0.018
Broken egg rate (%)	0.92 ± 0.03 ^b^	0.11 ± 0.01 ^a^	0.19 ± 0.01 ^a^	0.026
Soft shell egg rate (%)	0.12 ± 0.01	0	0.08 ± 0.01	0.177
Calcium deposition (%)	34.62 ± 1.36 ^b^	35.66 ± 1.28 ^a^	35.74 ± 1.17 ^a^	0.012

^a,b^ values within a row with different superscripts mean significant differences (*p* < 0.05). Note: Data are expressed as the means ± SE (standard error), *n* = 48. CON = basal diet; 2% FCH = 2% fermented Chinese herbs; 3% FCH = 3% fermented Chinese herbs.

**Table 9:** Effects of FCH on cholesterol and triglyceride deposition in laying hens.

Items		Treatment			*p*-Value
		CON	2% FCH	3% FCH	
Egg yolk	TC (mg/g)	12.38 ± 0.09 ^a^	11.04 ± 0.15 ^b^	10.65 ± 0.13 ^c^	<0.001
	TG (mg/g)	181.29 ± 8.36 ^a^	170.45 ± 7.29 ^b^	158.33 ± 5.15 ^c^	<0.001
Liver	TC (mg/g)	2.66 ± 0.02 ^b^	2.43 ± 0.01 ^ab^	2.27 ± 0.02 ^b^	0.035
	TG (mg/g)	11.53 ± 0.31 ^a^	10.18 ± 0.18 ^b^	9.64 ± 0.25 ^c^	0.013
	Bile acids (µmol/L)	33.18 ± 0.17 ^c^	39.64 ± 0.21 ^b^	43.47 ± 0.25 ^a^	<0.001

^a,b,c^ values within a row with different superscripts mean significant differences (*p* < 0.05). Note: Data are expressed as the means ± SE (standard error), *n* = 6 (liver) and 48 (eggs). CON = basal diet; 2% FCH = 2% fermented Chinese herbs; 3% FCH = 3% fermented Chinese herbs; TC = total cholesterol; TG = triglyceride.

## Data Availability

The original contributions presented in this study are included in the article; further inquiries can be directed to the corresponding author.
